# RNA-Binding Protein Rbm24 as a Multifaceted Post-Transcriptional Regulator of Embryonic Lineage Differentiation and Cellular Homeostasis

**DOI:** 10.3390/cells9081891

**Published:** 2020-08-12

**Authors:** Raphaëlle Grifone, Ming Shao, Audrey Saquet, De-Li Shi

**Affiliations:** 1 Developmental Biology Laboratory, CNRS-UMR7622, IBPS, Sorbonne University, 75005 Paris, France; raphaelle.grifone@sorbonne-universite.fr (R.G.); audrey.saquet@sorbonne-universite.fr (A.S.); 2 Shandong Provincial Key Laboratory of Animal Cell and Developmental Biology, School of Life Sciences, Shandong University, Qingdao 266237, China; shaoming@sdu.edu.cn

**Keywords:** RNA-binding protein, Rbm24, cell differentiation, alternative splicing, cytoplasmic polyadenylation, muscle, head sensory organ, lens, inner ear

## Abstract

RNA-binding proteins control the metabolism of RNAs at all stages of their lifetime. They are critically required for the post-transcriptional regulation of gene expression in a wide variety of physiological and pathological processes. Rbm24 is a highly conserved RNA-binding protein that displays strongly regionalized expression patterns and exhibits dynamic changes in subcellular localization during early development. There is increasing evidence that it acts as a multifunctional regulator to switch cell fate determination and to maintain tissue homeostasis. Dysfunction of Rbm24 disrupts cell differentiation in nearly every tissue where it is expressed, such as skeletal and cardiac muscles, and different head sensory organs, but the molecular events that are affected may vary in a tissue-specific, or even a stage-specific manner. Recent works using different animal models have uncovered multiple post-transcriptional regulatory mechanisms by which Rbm24 functions in key developmental processes. In particular, it represents a major splicing factor in muscle cell development, and plays an essential role in cytoplasmic polyadenylation during lens fiber cell terminal differentiation. Here we review the advances in understanding the implication of Rbm24 during development and disease, by focusing on its regulatory roles in physiological and pathological conditions.

## 1. Introduction

RNA-binding proteins (RBPs) play key roles in the post-transcriptional regulation of gene expression in a variety of biological processes. Vertebrate cells express hundreds to thousands of RBPs that display unique binding activity to their RNA targets and specific interaction with other protein partners. They control RNA metabolism at multiple levels, from alternative splicing, to transport, subcellular localization, stability, polyadenylation, and translation [1]. Thus, protein–RNA interactions are critical for maintaining the homeostasis of protein synthesis during early development and in adult life. Indeed, large-scale analyses of protein expression regulation have made the unprecedented finding that protein abundances within the proteome and mRNA levels within the transcriptome are entirely uncoupled in many conditions [2,3]. This highlights the importance of additional regulations by the ribonome, which embodies mRNAs and associated RBPs within a rich ribonucleoprotein (RNP) infrastructure in the cell [4]. Due to the crucial roles exerted by the processes downstream of transcription in the spatiotemporal control of gene expression, it is not surprising that a growing number of human diseases, such as neurodegenerative disorders and various types of cancers, are associated with RNAs and RBPs [5,6,7,8,9]. Therefore, RBPs may have the potential to be used as therapeutic targets in many diseased conditions [10,11,12].

Rbm24 (RNA-binding motif protein 24) is an evolutionarily conserved RBP that contains a single RNA recognition motif (RRM) at its N-terminal region [13,14,15]. It exhibits strongly restricted tissue-specific expression patterns during vertebrate development [16], and emerges as an important regulator of cellular differentiation and a potential factor implicated in human disease. Although no mutation of the human *RBM24* gene has been associated with any disease up today, deficiency in its expression level could be the cause of congenital disorders, such as cardiomyopathy, myopathy, or blindness, which affect the normal function of related tissues where crucial roles of this gene have been demonstrated in different animal models. It seems that vertebrate Rbm24 could be involved in nearly all aspects of post-transcriptional regulation. Most importantly, it functions as a key factor that regulates alternative splicing to establish the contractile function in developing cardiac and skeletal muscles [17,18,19], and plays an important role in cytoplasmic polyadenylation (CPA) to ensure the accumulation of crystallin proteins during lens fiber cell terminal differentiation [20]. There is thus accumulating evidence that Rbm24 acts as a multifaceted regulator to initiate cell differentiation through distinct mechanisms, which may vary in a tissue-specific and even a stage-specific manner. Moreover, Rbm24 presents almost the same characteristics in RNA and protein interactions, and often performs similar functions as the closely related ancient paralog Rbm38 (RNPC1) in regulating cell cycle progression and apoptosis [21,22], adding more complexity to its functional implication in various cellular processes. Mechanistic insights on how Rbm24 controls gene expression are beginning to be elucidated using in vivo and in vitro approaches. However, a number of important questions remain unanswered and await further investigation, such as its dynamic subcellular localization and tissue-specific function, the modulation of its activity through interaction with other partners, and its potential implication in human pathologies. In this review, we present recent advances in understanding the regulatory roles of Rbm24 in development and disease.

## 2. Rbm24 Functional Domains

**Figure 1:**
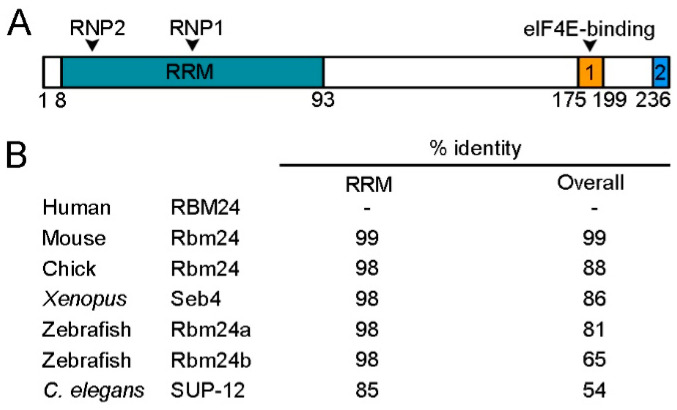
Rbm24 is a highly conserved RNA-binding protein (RBP). (**A**) Schematic representation of human RBM24 protein domains. The amino-terminal half contains a canonical RRM with two consensus RNP sequences (RNP1 and RNP2), and the C-terminal region contains two conserved domains (1 and 2), including an eIF4E-binding motif. Amino acid positions are indicated below. (**B**) Identity in the overall sequence and in the RRM between human RBM24 and Rbm24 proteins from other species. Human RBM24, NM_001143942.2; mouse Rbm24, NM_001081425.1; chick Rbm24, NM_001012863.3; *Xenopus laevis* Seb4, NM_001087526.1; zebrafish Rbm24a, NM_212865.1; zebrafish Rbm24b, NM_001328141.1; *C. elegans* SUP-12, NM_076273.5.

Rbm24 proteins display deep evolutionary conservation. The RRM is located at the N-terminal half and contains two characteristic consensus RNP sequences, RNP1 and RNP2 [23]. This domain shows extremely high levels of sequence identity among different species, ranging from a nematode to human (Figure 1).

In addition, it is almost identical to the RRM of Rbm38, such that both Rbm24 and Rbm38 prefers similar GU-rich sequences in target mRNAs [21,24]. Although the sequence outside the RRM is relatively divergent, at least two conserved domains can be identified in the C-terminal half of vertebrate Rbm24 and Rbm38 [25]. In particular, a motif close to the extreme C-terminus, which contains a serine residue (serine 181 in Rbm24 and serine 195 in Rbm38), interacts with eukaryote initiation factor 4E (eIF4E) and prevents it from binding to the 5’-cap of mRNAs [26,27]. However, at least in several cancer cell lines, phosphorylation of the serine residue by glycogen synthase kinase 3 (GSK3) prevents the interaction with eIF4E and converts Rbm24 or Rbm38 into an activator of mRNA translation [28]. A second conserved motif is the extreme C-terminus, whose implication in the Rbm24 function is not clear. The C-terminal half of Rbm24 also interacts with a number of other partners, but the exact motif implicated has not been determined yet, and the interaction is often assisted by the RRM. For example, both the N-terminal and C-terminal regions of Rbm24 are required for interaction with Stk38 kinase [29]. Although the N-terminal region of zebrafish Rbm24a does not directly interact with cytoplasmic polyadenylation element-binding protein 1b (Cpeb1b) and cytoplasmic poly(A)-binding protein 1l (Pabpc1l), its absence decreases the capacity of the C-terminal region to interact with these partners [20]. Thus, Rbm24 displays biochemical and functional conservation with respect to Rbm38, and its functionality in different cellular processes may be regulated through interaction with specific protein partners.

## 3. Rbm24 Displays Restricted Expression Patterns in Developing Vertebrate Embryos

**Figure 2:**
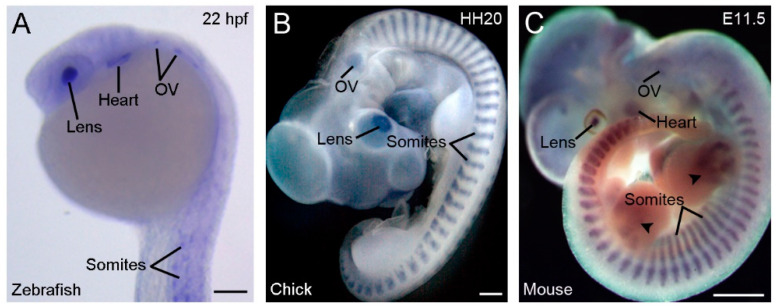
Conserved and restricted expression patterns of *Rbm24* gene in vertebrate embryos. (**A**) The expression of *rbm24a* in a zebrafish embryo at 22 hpf (hours post-fertilization). (**B**) A chick embryo at stage HH20 (3 days). (**C**) A mouse embryo at E11.5 [16]. Arrowheads indicate *Rbm24* expression in limb muscles. In all vertebrates, *Rbm24* is expressed in the somites, heart, lens, and otic vesicle (OV). Scale bars: (**A**) 100 µm; (**B**) 1 mm; and (**C**) 1 mm.

Another strikingly conserved aspect of vertebrate Rbm24 is the highly restricted developmental expression patterns (Figure 2).

**Figure 3:**
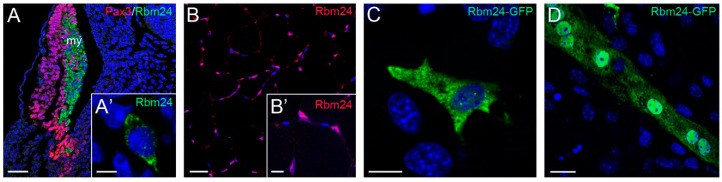
Dynamic subcellular localization of Rbm24 protein during muscle cell differentiation. (**A**) Immunofluorescence staining of a mouse embryonic section at E11.5 shows Rbm24 expression in differentiating myoblasts within the myotome (my), but not in Pax3-positive premyogenic progenitors. Some Pax3-expressing cells are intermingled with Rbm24-positive cells, but they do not yet express Rbm24 [16]. (**A’**) Higher magnification shows the punctate cytoplasmic staining of Rbm24 in a myoblast. (**B**) Immunofluorescence staining of Rbm24 in adult mouse muscles. (**B’**) Higher magnification shows nuclear localization of Rbm24. (**C**) Localization of Rbm24-GFP in the cytoplasm of murine C2C12 myoblasts. (**D**) Strong nuclear and weak cytoplasmic localization of Rbm24-GFP in differentiated C2C12 myotubes. Nuclei are stained with DAPI. Scale bars: (**A**) 50 µm; (**A’**) 10 µm; (**B**) 20 µm; (**B’**) 10 µm; (**C**) 10 µm; and (**D**) 10 µm.

During gastrulation, *Xenopus rbm24*, also called *XSeb4* or *MTG1*, is mainly expressed in the dorso-lateral mesoderm regions flanking the axial midline, which mostly overlap with the expression domains of *MyoD* [13,30]. During organogenesis, analyses by in situ hybridization and immunofluorescence staining indicate that Rbm24 is expressed in the somites, heart, and head sensory organs in different vertebrate embryos, including zebrafish, *Xenopus*, chick, and mouse [13,14,16,31,32,33,34,35,36]. Zebrafish genome contains two *rbm24* paralogs, *rbm24a* and *rbm24b*. The sites of *rbm24a* expression are identical as those of other vertebrate *Rbm24*, whereas *rbm24b* is mainly expressed in the somites and heart, but not in head sensory organs [33,34]. Within each tissue, Rbm24 is generally expressed in a subset of cells undergoing differentiation. The cellular localization of the Rbm24 protein in different tissues has been analyzed in more detail during mouse embryonic development [16,36]. Interestingly, Rbm24 displays dynamic subcellular localization during muscle cell development. In the myotome of mouse embryos and in murine C2C12 cells it is first accumulated in the cytoplasm of MyoD-positive myoblasts entering into the differentiation program, but not in Pax3-positive muscle progenitor cells, while in mature myotubes it is mainly present in the nucleus (Figure 3).

A cytoplasmic localization of the Rbm24 protein is also detected in different head sensory organs [36]. In the developing lens, it is restricted to differentiating fiber cells in the posterior and equatorial regions; in the otic vesicle, it co-localizes with Myo7A in inner ear hair cells; in the olfactory epithelium, it is expressed in fate-committed neuronal precursors and terminally differentiated olfactory receptor neurons. Thus, the predominant localization of Rbm24 in the cytoplasmic compartment of different cell types closely correlates with their entry into the process of differentiation. Detailed Rbm24 expression and subcellular localization will be further described in sections addressing its tissue-specific function. Herein we discuss the regulatory roles of Rbm24 and the consequences of its dysfunction in development and disease (Table 1).

## 4. Rbm24 Regulates Muscle Cell Development through Distinct Mechanisms

### 4.1. Rbm24 in Skeletal Myogenesis

The conserved expression of Rbm24 in the paraxial mesoderm of vertebrate embryos suggests that it may play a role in skeletal muscle development. Indeed, the *Xenopus rbm24* (*XSeb4*) gene has been shown to be a transcriptional target of MyoD and early B cell factor (EBF) family members of transcription factors, and relays the function of these factors during myogenesis [30,48]. In zebrafish, single knockdown of *rbm24a* or *rbm24b* indicates that they are both required for somitogenesis, but *rbm24a* seems to display a predominant activity [34]. It is unclear whether *rbm24a* and *rbm24b* cooperate in skeletal muscle development, and this question needs to be addressed by simultaneously inhibiting the function of both genes. In chick embryos, knockdown of *Rbm24* in the somites severely alters the expression of muscle-specific myosin, indicating that it is required for myogenic differentiation [16]. In *Rbm24* mutant mice, although no myofiber degeneration can be observed in skeletal muscles, there is a loss of M-bands in sarcomeres, which is accompanied by a reduced inclusion of muscle-specific exons in muscle cells [17]. This finding suggests that Rbm24 may be involved in skeletal muscle development through regulation of muscle-specific alternative splicing. It is consistent with the function of SUP-12, a *Caenorhabditis elegans* homolog of vertebrate Rbm24 that regulates alternative splicing of fibroblast growth factor (EGL-15) pre-mRNA in sex myoblast migration [49,50,51]. Thus, Rbm24 may represent an important conserved regulator of muscle-specific alternative splicing events in skeletal muscles. At present, the transcriptional control of muscle cell specification and differentiation has been relatively well documented [52,53], but the contribution of post-transcriptional regulation to muscle development remains largely unclear [54]. However, misregulation of several RBPs, including CELF (CUG-BP, Elav-like family), MBNL (Muscleblind-like) and RBFOX (RNA-binding forkhead box) families of proteins, disrupts muscle-specific alternative splicing and has been linked to skeletal muscle disease such as myotonic dystrophy [7,54]. Since skeletal muscle is one of the first tissues in which alternative splicing generates abundant contractile proteins from widely expressed genes has been identified [55], these observations provide mechanistic insights into muscle cell differentiation and function.

There are several lines of evidence suggesting that Rbm24 may not only function as a splicing factor, but also regulates skeletal myogenesis through other mechanisms. In vitro studies indicate that Rbm24 promotes myogenic differentiation of C2C12 cells by inducing cell cycle arrest upon binding to target mRNAs encoding cell cycle regulators [56], and by stabilizing *myogenin* mRNA through binding to its 3’-untranslated region [37]. These functionalities are entirely distinct from the regulation of alternative splicing. Thus, Rbm24 may display multiple or dynamic roles during the process of muscle cell development, which is consistent with its differential subcellular localization in myoblasts and multinucleated myotubes. Further in vivo analyses will be necessary to identify Rbm24-mediated stage-specific post-transcriptional mechanisms that trigger muscle lineage specification and differentiation.

### 4.2. Rbm24 is Required for Heart Development

Loss of Rbm24 severely impairs heart development in zebrafish and mouse embryos [14,17,20,33,38]. Knockdown or knockout of *rbm24a* in zebrafish is sufficient to cause severe cardiogenic defects [14,20,33]. Although *rbm24a* morphants or mutants have a beating heart, blood is only transported back and forth between the ventricle and atrium. This is due to the defective atrioventricular separation, which impairs blood circulation in the whole body [20]. In *Rbm24* homozygous mutant mice, heart malformations, including defective ventricular septum, reduced myocardial compaction, dilated atria and abnormal atrioventricular endocardial cushions, become evident at E10.5, these are followed by growth retardation at subsequent stages and embryonic lethality at E13.5 [17,38]. In addition to regulating early heart development, Rbm24 also plays a pivotal role in later cardiac sarcomerogenesis. Both in zebrafish and mice, loss of Rbm24 function causes disrupted Z-discs and sarcomere organization due to a decreased expression of sarcomeric proteins, a phenotype reminiscent of cardiomyopathy [14,17,19]. Although no defective alternative splicing is present in *Rbm24* heterozygous mutant mice, which are normal and viable, there is an increased sarcomere slack length and a lower myofilament passive stiffness in cardiomyocytes [57]. This raises a possibility that Rbm24 haploinsufficiency may influence disease penetrance in congenital heart disorders.

RNA-seq analyses have provided important insights on Rbm24-mediated post-transcriptional control of cardiac differentiation. Altered splicing of a large number of differentiation-related muscle genes has been identified in mouse embryos lacking Rbm24 function [17]. A majority of these altered splicing events is also present in zebrafish *rbm24a* mutants [20], suggesting a functional conservation of Rbm24-regulated alternative splicing in heart development. Further study on the consequences of Rbm24 deficiency in post-natal heart development using conditional knockout mice indicates a global disruption of alternative splicing events, which mostly affects those genes coding for sarcomere structure proteins involved in muscle contraction, including in particular *Ttn* [19]. It has been shown that the isoform switch of *Ttn* in cardiomyocytes is dependent on the function of Rbm20 [58], which possesses a single central RRM and regulates a large number of heart genes [59]. Moreover, mutations of both RBM20 and *TTN* genes in humans cause dilated cardiomyopathy [60,61,62]. This raises a possibility that human RBM24 may cooperate with RBM20 to regulate cardiac muscular functionality and may represent a new but rare gene associated with heart diseases [63]. Supporting the cooperation between Rbm24 and Rbm20 in heart development, it has been reported that the two proteins interact biochemically and bind to the same intronic region to promote the splicing of short *Enigma homolog* (*Enh*) splice variants, which encode LIM-less PDZ-LIM proteins that can prevent the hypertrophic growth of cardiomyocytes [64]. Similarly, in *Caenorhabditis elegans,* SUP-12 and its cofactors regulate muscle-specific alternative splicing through recognition of juxtaposed *cis*-elements on the target RNA to form a ternary complex [50,51,65].

Although defective alternative splicing represents an important event caused by loss of Rbm24 during heart development, other post-transcriptional regulatory processes are also affected. In particular, deficiency of Rbm24 in mice leads to aberrant activation of p53-dependent apoptosis in heart tissues, whereas overexpression of Rbm24 can inhibit p53 protein expression [38]. Mechanistically, in vitro studies suggest that in the absence of phosphorylation at serine 181 within the eIF4E-binding motif, Rbm24 prevents eIF4E from binding to p53 mRNA, thereby interfering with the cap-binding function of eIF4E and repressing p53 expression. In contrast, phosphorylation of this serine residue converts Rbm24 into an activator of p53 expression [38]. These observations suggest that Rbm24 may be involved in regulating p53 activity during heart development in a phosphorylation-dependent manner. Since p53 induces apoptosis in disease- or aging-related failing heart [66], Rbm24 may exert a protective role to reduce the incidence of heart failure. Thus, it would be interesting to examine this possibility and to analyze the regulation of Rbm24 activity by phosphorylation during heart development. Consistent with its implication in regulating post-transcriptional processes downstream of alternative splicing, immunofluorescence staining indicates that Rbm24 protein is abundantly accumulated in the cytoplasm of mouse cardiomyocytes at E11.5 [16]. The distinct roles of Rbm24 during cardiac muscle development imply that it could be involved in various aspects of post-transcriptional regulation. Indeed, RNA immunoprecipitation assay followed by a microarray analysis in cardiac myoblast cell lines suggests that Rbm24 binds to, and regulates numerous targets through distinct mechanisms, including mRNA stability, alternative splicing and transcriptional initiation, and mRNA translation [67]. This highlights the importance of the Rbm24-organized RNA regulon in coordinating and stabilizing the expression of structural and functional genes in cardiac muscle [68].

### 4.3. Dynamic Subcellular Localization and Function of Rbm24

Analysis of Rbm24 subcellular localization provides further support on the possibility that it performs multiple and dynamic functions during muscle cell development. Within the myotome of mouse embryos at E11.5, the Rbm24 protein only accumulates in the cytoplasm of MyoD-positive myoblasts engaged in the differentiation program, but not in Pax3-expressing premyogenic progenitors [16], which represent a proliferating population of muscle stem cells [69]. This implies that Rbm24 functions in the cytoplasmic compartment during the differentiation step of muscle development, but its post-transcriptional regulatory roles remain to be explored. Interestingly, and consistent with its function in regulating alternative splicing of those mRNAs encoding muscle-specific contractile proteins, the localization of the Rbm24 protein in the myofibers of mouse adult muscles is only restricted to the nucleus. This translocation as a function of cell differentiation state can also be observed in murine C2C12 cell line expressing Rbm24-GFP. The fusion protein is first expressed in the cytoplasm of mononucleated myoblasts, and then accumulates in the nucleus of multinucleated myotubes (Figure 3). These in vivo and in vitro observations suggest that Rbm24 may exert distinct post-transcriptional activities in differentiating myoblasts and terminally differentiated myofibers. Thus, there is a possibility that its regulatory roles on muscle cell differentiation and muscular functionality may be cell-type specific, which may depend on its subcellular localization and the presence or absence of its co-factors.

It is likely that the cytoplasmic localization of Rbm24 in myoblasts functions to regulate the stability and/or translation of mRNAs encoding muscle differentiation-promoting factors required for early differentiation steps, such as myogenin [37], thereby switching them into the differentiation program. As cells become further differentiated during later steps of myofibrillogenesis, Rbm24 accumulates in the nucleus to promote the expression of muscle structural and contractile proteins through alternative splicing. Consistent with its role in regulating the transition from embryonic cell pluripotency to differentiation, overexpression of Rbm24 is able to induce the specification and trigger the differentiation of mouse embryonic stem cells into cardiomyocytes [70]. Thus, the dynamic subcellular localization of Rbm24 argues that it may regulate multiple steps of myogenic differentiation through distinct post-transcriptional mechanisms. However, the shift of Rbm24 protein from cytoplasm to the nucleus during muscle differentiation raises an important question as to how it is shuttling between these subcellular compartments. Whether this depends on the presence or absence of a signal/co-factor that also displays dynamic changes during muscle cell maturation? Interestingly, a similar cytoplasm to nucleus translocation has been observed for MBNL in post-natal skeletal muscle [71], although its cytoplasmic function during muscle development is not clear [7]. Therefore, the significance of the dynamic characteristics of RBP subcellular localization during muscle development is intriguing and merits further investigation.

## 5. Rbm24 in Head Sensory Organ Development

### 5.1. Rbm24 Regulates Lens Fiber Cell Differentiation

Although much attention has been focused on the Rbm24 function in cardiac and skeletal muscle cell differentiation, several recent works have revealed an interesting expression profile and an important role of this protein during the development of vertebrate head sensory organs [20,35,36,39,72]. In particular, it has been shown that Rbm24 is required for lens fiber cell terminal differentiation in zebrafish and in mice, providing further evidence for an essential regulatory role mediated by RBPs in vertebrate lens development. Indeed, several RBPs have been shown to be required for eye development in humans or animal models [73]. For example, mutations of the Tudor domain protein TDRD7 in humans cause cataract formation [74], illustrating a critical implication of post-transcriptional events in lens morphogenesis.

**Figure 4:**
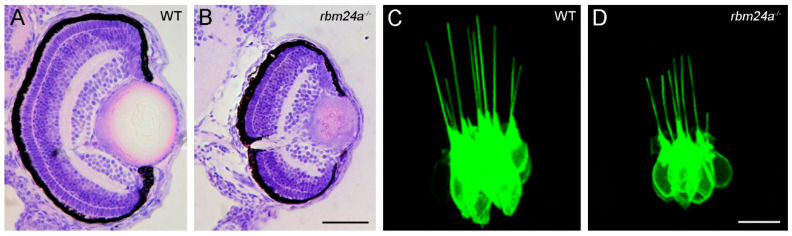
Loss of Rbm24a function impairs lens transparency and affects inner ear hair cell development in zebrafish. (**A**,**B**) Histological sections of ocular tissues at the level of the optic nerve compares lens differentiation between a wild-type (WT) sibling (**A**) and an *rbm24a* mutant (**B**) at 3 dpf (days post-fertilization). The sections were stained by hematoxylin and eosin. Loss of Rbm24a disrupts lens differentiation and causes cataract formation, but has no effect on retina differentiation. The microphthalmia phenotype and defective lens fiber cell denucleation are secondary consequences due to impaired blood circulation [20]. (**C**,**D**) Confocal microscopic analyses compare hair cell development and organization in the lateral crista of the zebrafish inner ear from a wild-type (WT) sibling (**C**) and an *rbm24a* mutant (**D**) at 3 dpf, under the *Tg(pou4f3:GAP-GFP)* transgenic background [40]. Scale bars: (**A**,**B**), 50 µm; (**C**,**D**) and 10 µm.

Lens transparency is established by abundant accumulation of crystallin proteins and denucleation in lens fiber cells [75]. Loss of Rbm24a function in zebrafish directly prevents the efficient translation of *crystallin* mRNAs into functional proteins, and indirectly affects lens fiber cell denucleation due to impaired blood circulation [20]. As a consequence, *rbm24a* mutant embryos develop a severe cataract phenotype (Figure 4A,B).

Detailed examination of the Rbm24 protein expression in mouse embryos indicates that it not only shows specific expression in differentiating lens fiber cells, but also accumulates in the cytoplasm of these cells [36]. During zebrafish development, the expression of *rbm24a* in the lens placode can be detected around 17 hpf (hours post-fertilization), several hours before the initiation of primary fiber cell differentiation. At early stages of lens development, Rbm24a may participate in the post-transcriptional regulation of several genes involved in the specification of the lens placode, such as *Pax6* and *Sox2*. Consistently, Rbm24a deficiency results in decreased stability of *Sox2* mRNA [39]. As lens fiber cell differentiation proceeds, *rbm24a* exhibits highly localized expression patterns [20]. After 24 hpf, it is progressively restricted to the posterior and equatorial regions of the lens mass, where differentiation of primary fiber cells and initial formation of secondary fiber cells take place [76]. This dynamic expression of *rbm24a* in lens fiber cells makes it a strong candidate in regulating lens fiber cell terminal differentiation. Indeed, the localization of Rbm24a protein in the cytoplasm of differentiating lens fiber cells before and after nuclear degradation suggests that it should regulate post-transcriptional events other than alternative splicing.

Importantly, The RRM of Rbm24a binds to a wide spectrum of lens-specific mRNAs that encode either small heat shock proteins or lens structural proteins, and the C-terminal region interacts with members of the CPEB and PABPC families [20]. CPEB and PABPC are key components of the CPA complex that regulates poly(A) tail elongation of nuclear exported mRNAs [77,78,79]. They are required for translational activation in a variety of processes, including oogenesis and early embryonic development [80,81]. Loss of Rbm24a specifically reduces the poly(A) tail length of many *crystallin* mRNAs, which severely prevents the accumulation of lens transparent proteins and causes cataract formation [20]. The demonstration that Rbm24a may function in the CPA complex is significant in understanding the regulatory mechanism underlying mRNA translational activation to switch and maintain lens fiber cell differentiation. Indeed, when transcriptional contribution declines during denucleation of differentiating lens fiber cells, a post-transcriptional mechanism that promotes the efficient translation of lens-specific mRNAs will be necessary for the production of high amounts of transparent proteins. Zebrafish and mouse Rbm24 is expressed at the right time and place to play a critical role in this process, suggesting that similar post-transcriptional mechanisms control lens development in vertebrates. The importance of post-transcriptional regulation after lens fiber cell denucleation has been also demonstrated for other aspects of lens morphogenesis. Analysis of TDRD7 function in mice indicates that it is required for maintaining cytoskeletal organization and lens fiber cell morphology by regulating the expression of heat shock protein HSPB1 [82]. Moreover, members of the PABPC family are ancient paralogs of Rbm24, and they are coexpressed in different tissues. For example, zebrafish Pabpc4 also displays strongly localized expression in the somites and lens primordium during early development [83]. This correlative evidence further supports a potential interaction between these RBPs in regulating CPA during lens differentiation. Nevertheless, it remains to be determined how Rbm24 functions precisely within the CPA complex to control mRNA translation in lens fiber cells, and whether it regulates cell differentiation in other tissues through the same mechanism.

### 5.2. Rbm24 and Rbm38 in Retinal Differentiation

Immunofluorescence staining indicates that the mouse Rbm24 protein is expressed in the optic vesicle at E9.5 to E11.5 [39], but not at later stages [36,39]. Although both zebrafish and mouse *Rbm24* mutants display the microphthalmia phenotype [20,39,72], this seems to be indirectly caused by the absence of blood supply because the rescue of blood circulation in the zebrafish mutants prevents microphthalmia but not cataract formation [20]. Thus, it is at present still unclear whether Rbm24 plays a role in retinal development. Nevertheless, XSeb4R (Rbm38) has been shown to regulate retinal neuronal differentiation during *Xenopus* development. It is expressed in retinoblasts and undifferentiated post-mitotic neurons. Gain-of-function of XSeb4R promotes neural differentiation, whereas loss-of-function causes an increased number of glial cells [25]. The post-transcriptional mechanism in retinogenesis mediated by XSeb4R is not clear. However, given the biochemical and functional conservation between Rbm24 and Rbm38, there is a possibility that it may bind to, and stabilize *Sox2* mRNA in the gene regulatory network of the optic vesicle [39].

### 5.3. Rbm24 Is Required for Inner Ear Hair Cell Development

The strongly restricted expression of vertebrate Rbm24 in the inner ear also makes it a potential post-transcriptional regulator of hair cell differentiation and regeneration. In the cristae of the neonatal mouse vestibular system, in situ hybridization analysis shows that the expression of the *Rbm24* gene in a subset of hair cells is directly regulated by the transcription factor Atoh1 [35]. In the embryos at E14.5, Rbm24 protein co-localizes with Myo7A in mechanosensory cells of the auditory and vestibular systems [36]. These expression patterns suggest that Rbm24 may function downstream of hair cell fate specification, and may participate in hair cell differentiation or function. Nevertheless, it is at present still unclear whether and how Rbm24 regulates hair cell development in mammalian embryos, and generally, post-transcriptional regulatory mechanisms underlying hair cell development and homeostasis remain largely elusive, mostly due to the lack of functional studies on related gene regulatory networks.

Zebrafish have become particularly attractive for understanding otic development and human diseases affecting hearing function [84,85]. The transcripts of zebrafish *rbm24a* can be detected in the anterior and posterior maculae at least at 14 hpf [14]. Single cell RNA-seq analysis indicates that *rbm24a* expression dynamically changes from non-cycling progenitors to differentiated hair cells, suggesting that Rbm24a protein may participate in the differentiation program [86]. A recent study has established a functional requirement of Rbm24a for hair cell development [40]. Rbm24a deficiency does not affect the specification of the otic vesicle, however, it prevents the differentiation of hair cells in the anterior, lateral and posterior cristae corresponding to the sensory patches for each of the three semicircular canals. This is associated with a disorganization of hair cells and a reduced height of their kinocilia that are involved in the morphogenesis of the hair bundle and in mechanotransduction (Figure 4C,D). Loss of Rbm24a also impairs the organization of hair cells in the neuromasts of the posterior lateral line system. RNA-seq and qRT-PCR analyses reveal a reduced expression level of several zebrafish orthologs of human deafness genes, such as *smpx*, *gsdmeb* and *otofa* in *rbm24a* mutants [40]. Mutations of these genes in humans have been associated with X-linked deafness-4, autosomal dominant nonsyndromic deafness 5 and sensorineural hearing loss, and autosomal recessive nonsyndromic deafness 9, respectively. Inhibiting the function of these genes in zebrafish produces similar defects in the developing ear as observed in *rbm24a* mutants [87,88]. Consistently, loss of Rbm24a causes hearing and balancing deficits, demonstrating its functional requirement for hair cell differentiation and/or function [40]. Since no defective alternative splicing of these genes has been detected by RNA-seq analysis in *rbm24a* mutants [20], how Rbm24a regulates their expression remains unclear, but it is likely that these mRNAs display reduced stability in the absence of the Rbm24a function. Further study on the regulatory hierarchy implicating Rbm24 in the development of inner ear sensory receptors will help to understand the post-transcriptional mechanism operating in this lineage.

### 5.4. Rbm24 and Differentiation of Olfactory Sensory Neurons 

The functional implication of Rbm24 in the differentiation of sensory neurons within the olfactory epithelium has not been investigated to date. Immunofluorescence staining of mouse embryonic sections indicates that Rbm24 expression is restricted to differentiating neurons [36]. At E10.5 and E11.5, it mainly accumulates in the cytoplasm of fate-committed immediate neuronal precursors (INPs) and terminally differentiated olfactory receptor neurons (ORNs) within the nasal pit, suggesting that it may promote neurogenic differentiation by regulating the translation of target mRNAs. At a later stage (E14.5), Rbm24 protein is strongly expressed in basal neural stem cells and weakly expressed in differentiating olfactory neurons. These observations raise the possibility that Rbm24 may be involved in maintaining the progenitor population and/or switching their progression toward more committed olfactory neuronal cells. During olfactory epithelium development, it is well established that transcriptional and epigenetic controls of spatiotemporal gene expression determine the formation of olfactory sensory neurons and non-neuronal cell types [89]. However, there is also evidence that mRNAs encoding olfactory receptors in neural lineages are protected from degradation, suggesting a requirement of RBPs-mediated post-transcriptional regulatory mechanisms in their stabilization [90]. Thus, it is worth analyzing whether and how neuronal differentiation in the olfactory epithelium is affected following the loss of Rbm24 function, in order to elucidate the molecular and cellular mechanisms underlying neurogenesis of the olfactory system. Since the expression of Rbm24 in the olfactory epithelium is only examined in mice, it is also of interest to see whether this expression pattern is conserved in other vertebrates.

## 6. Rbm24 in Embryonic Germ Layer Formation

In *Xenopus* and zebrafish, *XSeb4* and *rbm24a* are expressed as maternal transcripts [13,14]. However, their spatial localizations within the cleavage stage embryos and their maternal functions are not clear and await further investigation. Knockdown of *XSeb4* by injecting translation-blocking morpholino oligonucleotide into fertilized *Xenopus* eggs has been shown to inhibit the expression of mesoderm genes at the early gastrula stage, such as the pan-mesoderm marker *brachyury* [30]. Nevertheless, it is unclear whether this represents a direct effect or an indirect consequence due to the disruption of upstream events, such as maternal regulators of the *brachyury* gene. Interestingly, several studies have focused on the maternal function of XSeb4R (Rbm38). It has been shown that XSeb4R participates in germ layer formation by binding to the vegetally localized maternal *VegT* mRNA and positively regulating its stability and translation [91]. Since VegT is a critical T-box transcription factor required for the inductive interaction and germ layer specification [92], the regulation of its expression by Rbm24-related proteins may be important for the initial formation of endoderm and mesoderm tissues. Maternal *XSeb4R* transcripts are also enriched in the ectoderm, where the translated protein binds to, and promotes the translation of maternal *Sox3* mRNA [93]. The importance of maternal XSeb4R functions in promoting germ layer-specific translational activation of maternally stored mRNAs demonstrates a major contribution of post-transcriptional regulation in setting up maternal inputs to trigger zygotic developmental programs. On the other hand, the spatiotemporal activation and function of zygotic genes also depend on RBPs-mediated clearance of maternal mRNAs [94]. Therefore, whether Rbm24-related proteins may be also implicated in this process merits an investigation.

It is well established that maternal RBPs are critical mediators of translational regulation in the early embryos [95]. Before meiotic maturation, the translation of many maternal mRNAs is repressed partly due to the lack of an appropriate length of poly(A) tails [95,96]. Upon fertilization and during cell-cycle progression, CPA mediated by members of the CPEB and PABPC families plays a critical role in the stabilization and translational activation of oocyte-stored mRNAs by elongating their poly(A) tails [95,96,97]. Inhibition of CPA prevents transcription of zygotic genes and results in failure of maternal to zygotic transition and delay of developmental progression [98]. Since Rbm24 interacts with Cpeb1b, Pabpc1l, and eIF4E [20,38], which are conserved mRNA interactors required for the translational activation of maternal mRNAs [95], it is potentially involved in the regulation of CPA. Thus, it will be of interest to examine whether maternal Rbm24 regulates the dynamics of CPA for differentially activating or repressing the translation of germ layer-specific maternal mRNAs coding for important mediators of inductive interaction. It is equally important to understand how the spatiotemporal activity of Rbm24 is regulated by other factors in this process.

## 7. Potential Implication of Rbm24 in Disease

### 7.1. A possible Tumor Suppressor Activity of Rbm24 and Rbm38

RBPs regulate gene expression at multiple levels, and they are implicated in a large variety of biological processes through versatile interactions with RNAs. Alterations in protein–RNA interactions have been causally related to the occurrence of various cancers in humans [9]. Since Rbm24 protein contains a canonical RRM that binds to the GU-rich ligand present in a wide spectrum of target mRNAs [21,24], it would be not surprising that inappropriate regulation of its expression or function in humans perturbs the homeostasis of protein synthesis and leads to cancer development. There is accumulating evidence that Rbm24 displays tumor repressive activity. In several cancer cell lines, such as MCF7 and HaCaT cells, both Rbm24 and Rbm38 are transcriptional targets of the tumor suppressor p53, and their overexpression increases the stability of *p21* mRNA [99,100]. Thus, they can function to induce cell cycle arrest and to prevent tumor cell proliferation. However, this p53-induced anti-tumor activity of Rbm24 and Rbm38 needs to be reconciled with their oncogenic potential, which is related to their repressive function on *p53* mRNA translation and p53-dependent apoptosis [26,38]. Since serine phosphorylation in the eIF4E-binding motif of Rbm24 and Rbm38 converts them into an activator of p53 expression [28], it will be intriguing to compare their phosphorylation status between normal and tumor cells, in order to understand how they regulate mRNA translation in pathological processes. Another enigma is the significance of Rbm24 and Rbm38 in preventing p63-mediated tumor suppression because they have been shown to destabilize *p63* mRNA in overexpression experiments [101,102]. Again, this may be modulated by serine phosphorylation. An increase in the level of phosphorylated Rbm38 by GSK3ß enhances p63 expression, but this seems to be dependent on its dissociation with the Ago2-miR203 mRNA decay complex [103]. Thus, the functionality of Rbm24 and Rbm38 in tumorigenesis seems to be regulated by the activity of their associated kinases.

In other situations, the tumor suppressor activity of Rbm24 does not seem to be dependent on its phosphorylation. A recent study indicates that in prostate cancer, the microRNA miR-106a-5p promotes cell proliferation by inhibiting Rbm24 expression, and overexpression of wild-type Rbm24 is able to inhibit tumorigenesis [41]. It has been also shown that in nasopharyngeal carcinoma, the expression of Rbm24 is reduced, which leads to the down-regulation of miR-25 that has the ability to suppress cell proliferation by targeting the pro-oncogenic lncRNA MALAT1. Increasing the expression of Rbm24 can suppress cellular proliferation, migration and invasion [42]. These observations thus demonstrate an inhibitory effect of Rbm24 in tumorigenesis. A further understanding of the functional consequences of Rbm24 and Rbm38 in regulating cancer-related gene expression should help to define novel therapeutic strategies for modulation of their activity. Moreover, deciphering the detailed mode of RNA-binding by the conserved RRM of Rbm24 and Rbm38 could provide a basis for generating engineered mutants to modulate their interactions with RNA targets [104,105]. It is also important to understand how the expression and activity of Rbm24 or Rbm38 are dysregulated in cancer cells. In this regard, it has been shown that in hepatocellular carcinoma, the expression of *Rbm24* and several cancer-related genes is reactivated due to increased demethylation of their enhancers [106], suggesting that epigenetic modulation of Rbm24 expression in tumor cells may have the potential to prevent aberrant cell proliferation.

### 7.2. Interaction Between Rbm24 and microRNAs to Maintain Cellular Homeostasis

It has become increasingly evident that the interplay between RBPs and microRNAs represents a new level of complexity in the regulation of gene expression in various cellular processes, which either promotes or represses cancer development [107]. As in prostate cancer and nasopharyngeal carcinoma, Rbm24 also interacts with microRNAs in other tissues to maintain cellular homeostasis. The MIR143 host gene (MIR143HG) is a precursor of miR-143 and miR-145, and is overexpressed in colon tissues of patients with Hirschsprung disease (HSCR). Rbm24 forms a negative feedback loop with MIR143HG, and regulates its expression and stability. Thus, an alteration of this balance could lead to defective proliferation or migration of enteric neural crest cells and gangliogenesis in the gut of HSCR patients [43]. Interestingly, the expression of miR-125b-5p is enriched in heart valves. It binds to, and inhibits the expression of *Rbm24* mRNA [108]. Since loss of Rbm24 leads to heart valve defects, this raises a possibility that an imbalance between miR-125b-5p and Rbm24 may disrupt heart development and function. Similarly, miR-222 prevents myogenic differentiation and myoblast fusion. It exerts these effects partially by inhibiting Rbm24 expression [109]. These observations suggest that Rbm24 interacts with microRNAs in post-transcriptional regulatory interactions with potential contributions to muscle physiopathology.

### 7.3. Rbm24 in Congenital Disorders and Infection Diseases

A more plausible role of Rbm24 in human disease is its ability to promote U1 snRNP recognition of the mutated, but not the wild-type 5’ splicing site of *IKBKAP* gene (*inhibitor of κ light polypeptide gene enhancer in B cells, kinase complex-associated protein*) in familial dysautonomia (FD), an autosomal recessive disease [44]. The intronic 5’ splicing site mutation of *IKBKAP* gene in FD disrupts the splicing of exon 20, but this produces altered spliced mRNA product only in a tissue-specific manner, affecting essentially sensory and autonomic nervous system [110]. Rbm24 binds to an element downstream of the mutated splicing site and functions as a splicing enhancer [44]. Thus, its tissue-specific expression may help to correct the abnormal splicing of the *IKBKAP* gene and to reduce the penetrance of this genetic disorder, making it a potential therapeutic target for FD. This observation highlights the importance of regulated Rbm24 activity in maintaining cellular function and homeostasis. Indeed, as presented above, loss of Rbm24 in mice causes cardiomyopathy [14,17,19], but overexpression of Rbm24 has been shown to induce cardiac fibrosis in the mouse model by promoting collagen synthesis [111]. Moreover, Rbm24 not only regulates the expression of the organism’s own genes, several in vitro analyses suggest that it can also function as a host factor to participate in the translation, replication, and pregenomic RNA packaging of types B and C of hepatitis viruses [45,46,47]. Thus, we are just beginning to perceive the involvement of Rbm24-mediated post-transcriptional events in the etiology of various cellular dysfunctions. It is clear that the increasing association of Rbm24 with various human diseases requires a more comprehensive mechanistic understanding of its post-transcriptional regulatory functions.

## 8. Conclusions and Perspectives

Rbm24 emerges as a major post-transcriptional regulator in the switch of cell differentiation during vertebrate development. It represents a multifunctional RBP that orchestrates different gene expression circuits in a tissue-specific and stage-specific manner. There is also accumulating evidence that it maintains the homeostasis of protein synthesis in adult tissues. Thus, it is not surprising that dysfunction of Rbm24-mediated post-transcriptional regulation of gene expression affects early development and leads to the occurrence of various diseases. Many intriguing questions regarding Rbm24 function during development and disease remain unanswered, such as its maternal activity in translational regulation, the mechanism underlying its dynamic subcellular localization and function during muscle cell differentiation, the modality of its implication in CPA, and its association with human diseases. At present, no human congenital disorders have been directly linked to Rbm24 mutations, probably due to the lethality resulted from impaired heart development and blood circulation. In this regard, it would be also of interest to identify and understand the function of those *cis*- and *trans*-regulatory elements that control the spatiotemporal expression of Rbm24, which may be disrupted in human diseases. In addition, due to the importance of RBP autoregulation to maintain protein homeostasis or to switch cell fate changes [112], it would be important to understand how Rbm24 self-regulates to maintain cellular homeostasis or to initiate cell differentiation during development. Furthermore, vertebrate Rbm24 also interacts and cooperates with other closely related RBPs, such as Rbm20 and Rbm38, in the regulation of cell differentiation and function [56,64]. Mutations of RBM20 have been clearly associated with human congenital disorders, such as cardiomyopathy [60,61], while inappropriate expression of RBM38 is associated with tumorigenesis [26]. Thus, understanding the mechanisms by which Rbm24 cooperates with other factors in the regulation of gene expression should help to develop approaches for therapeutic manipulation.

## Figures and Tables

**Table 1:** Potential Rbm24 functions in development and disease.

Tissue or Disease	Post-Transcriptional Regulation	Role in Development or Disease
Skeletal muscle	Muscle-specific pre-mRNA splicing, mRNA stability	Myogenic differentiation, somitogenesis, and sarcomere organization [16,17,30,34,37]
Cardiac muscle	Muscle-specific pre-mRNA splicing, mRNA stability and translation	Heart development, sarcomere assembly, and cardiac contractility [14,17,19,33,38]
Lens	Cytoplasmic polyadenylation, mRNA stability	Lens fiber cell differentiation, and lens transparency [20,39]
Inner ear/Neuromasts	mRNA stability	Hair cell morphogenesis and differentiation [40]
Olfactory epithelium	Unknown (cytoplasmic localization in neuronal cells)	Unknown
Blastula/Gastrula	Unknown	Germ layer formation [30]
Prostate cancer	Interaction with miR-106a-5p	Inhibition of tumorigenesis [41]
Nasopharyngeal carcinoma	Interaction with miR-25	Inhibition of cell proliferation, migration and invasion [42]
Hirschsprung disease	Interaction with MIR143HG	Proliferation or migration of enteric neural crest cells, and gangliogenesis [43]
Familial dysautonomia	Increased recognition of the mutated 5’ splicing site in *IKBKAP* gene by U1 snRNP	Possible protective role against the aberrant splicing of the mutated gene [44]
Hepatitis A and B	Pregenomic RNA packaging and replication	Possible host factor for the viruses [45,46,47]
